# Specular Microscopic Corneal Endothelial Cell Changes following Uneventful Phacoemulsification in Patients with Gout

**DOI:** 10.1155/2022/1153504

**Published:** 2022-04-06

**Authors:** Mortada Abozaid, Rana Saad-eldin, Mahmoud Farouk, Mohamed Anbar, Ehab Wasfi

**Affiliations:** ^1^Department of Ophthalmology, Sohag University, Sohag, Egypt; ^2^Department of Rheumatology and Rehabilitation, Sohag University, Sohag, Egypt; ^3^Department of Ophthalmology, Assiut University, Assiut, Egypt

## Abstract

**Purpose:**

To assess the effects of phacoemulsification on the corneal endothelium in patients with gout and age-related cataracts.

**Methods:**

Eighty-eight patients who underwent uneventful phacoemulsification for age-related cataracts were included in this retrospective comparative study. The patients were divided into two groups: group A included 31 patients with gout and tight control of uric acid levels and group B included 57 patients without gout or any other systemic disease. All patients completed follow-up assessments over 6 months, and the two groups were compared in terms of changes to their specular microscopic values.

**Results:**

Both groups showed marked improvement in uncorrected and best-corrected visual acuity at the end of the follow-up period. Corneal endothelial cell loss was more severe in group A than in group B, with a mean difference of 221.35 ± 43.87 in group A and 169.88 ± 52.67 in group B at the sixth month (*p* < 0.001). The difference between the two groups in terms of other specular microscopic values did not reach statistical significance.

**Conclusion:**

Patients with gout are more prone to corneal endothelial cell damage after phacoemulsification than those without gout. To confirm these results, future studies with a prospective design and longer durations of follow-up are needed.

## 1. Introduction

Gout is an inflammatory condition caused by metabolic dysfunction, which can lead to elevated body uric acid levels (≥6.8 mg/dL), in turn triggering deposition of monosodium urate (MSU) crystals in different tissues, especially the joints. This condition mostly affects postmenopausal women and middle-aged men [1–4]. Changes in lifestyle and increasing rates of obesity are probably responsible for the doubling of gout prevalence over the past couple of decades.

Tophi are lesions resulting from the deposition of MSU crystals in the eye, especially in the ocular adnexa and anterior segment. Patients with tophi usually present with bilateral acute or chronic conjunctivitis and hyperaemic vessels. They may also develop cataracts caused by hyperuricaemia itself or from drugs used in the treatment of gout, such as steroids and allopurinol. Other ocular manifestations of gout include dry eye syndrome, uveitis, scleritis, and glaucoma [5–7]. Gout is also known to be associated with endothelial dysfunction of the blood vessels and renal tubules, and high levels of uric acid in the anterior chamber may accelerate apoptosis of the corneal endothelial cells, thus increasing corneal thickness [8–10].

The frequency of phacoemulsification cataract surgery is increasing these days because of its several advantages, including rapid rehabilitation, fewer complications, and reasonable cost. However, one of the primary limitations of this procedure is the potential for postoperative damage to the corneal endothelium. The integrity of the corneal endothelium is very important for dehydration of the corneal stroma and clarity of the cornea. Any compromise in the barrier function or active pump mechanism of the endothelium can result in corneal oedema [11]. Severe damage to the endothelium after phacoemulsification may cause corneal decompensation with a marked drop in vision, which may necessitate corneal transplantation and its related complications. Considering the decline in endothelial function with advancing age, great attention should be paid to the corneal endothelium before and during cataract surgery in elderly people, especially those with systemic comorbidities, to achieve the main goal of surgery, which is an improvement of vision [12, 13].

The aim of this study was to evaluate the effect of uneventful phacoemulsification on the corneal endothelium of patients with gout and compare the results with those obtained in a group of patients without gout or any other systemic disease. Endothelial cell density (ECD), coefficient of variation (CV), hexagonality (HEX), and central corneal thickness (CCT) were the parameters compared between the two groups.

## 2. Patients and Methods

This retrospective comparative study was conducted using the charts of 88 patients who had undergone uneventful phacoemulsification for visually significant age-related cataracts at the Ophthalmology Department of Sohag University Hospital and Assiut University Hospital. The patients were treated in collaboration with the respective rheumatology departments. The Ethics Committee of the Sohag Faculty of Medicine approved the study (approval number: soh-Med-21-03–14), which adhered to the tenets of the Declaration of Helsinki, and written informed consent was obtained from all patients before surgery. The study eyes were divided into two groups: group A, which included 31 eyes from 31 patients with age-related cataracts and gout defined according to the American College of Rheumatology 2015 gout classification criteria [14], and group B, which included 57 eyes from 57 patients with age-related cataracts without gout or any other systemic disease. The inclusion criteria included cataracts with nuclear grades N2 or N3 according to the Lens Opacities Classification System III [15] and a normal fundus and normal intraocular pressure (IOP). In contrast, we excluded patients with corneal pathology or a low endothelial cell count (<2000 cells/mm), contact lens wear, poor pupillary dilatation, pseudoexfoliation syndrome, glaucoma, uveitis, history of eye trauma, surgery, or any systemic disease that could have affected the endothelium, such as diabetes mellitus.

All study patients underwent a complete ophthalmic examination before and after surgery, including measurements of uncorrected visual acuity (UCVA) and best-corrected visual acuity (BCVA) in the logarithm of minimum angle of resolution (logMAR) values, evaluation of manifest refraction, slit-lamp and fundus examinations, IOP measurement using an applanation tonometer, noncontact specular microscopy using CEM-530 (Nidek, Gamagori, Japan), and measurement of the axial length (AL), anterior chamber depth (ACD), corneal power (K1, K2, K_average_, and keratometric cylinder), and intraocular lens (IOL) power using IOLMaster^®^ 500 (Carl Zeiss Meditec AG, Jena, Germany).

### 2.1. Sample Size Calculation

In the absence of previous similar studies, we relied on a pilot study to calculate the sample size. Using a small set of preliminary data collected from five patients with gout (group 1) and 10 nongout patients (group 2), the mean and standard deviation of the difference between the preoperative and 6-month postoperative values of the endothelial cell density were calculated to be 209.4 ± 110.2 in group 1 and 143.7 ± 89.3 in group 2. Then, a G^∗^ Power software (Dusseldorf University, Germany) was used to calculate the effect size which is utilised in a priori analysis together with an alpha error of 0.05 and a power of 0.8 to detect the sample size required to reveal a significant change in the endothelial cell density. The allocation ratio was set as 2 to increase the statistical confidence. We found that the study should include 30 patients with gout and 60 patients without gout.

### 2.2. Surgical Procedures

All patients were operated on by two surgeons (MA and EW) who conducted stop-and-chop phacoemulsification using the Infiniti^®^ Ozil continuous phacomachine (Alcon, Fort Worth, Texas, USA) with intelligent phacoemulsification (IP) software. To obtain adequate pupillary dilation before surgery, a topical mixture of cyclopentolate hydrochloride (1%) and phenylephrine hydrochloride (10%) was instilled into the eye every 10 min for 30 minutes. The surgery started with instillation of the topical anaesthetic benoxinate hydrochloride 0.4% into the conjunctival sac, which was followed by retrobulbar and facial nerve blocks using 2% lidocaine HCl injection.

Two paracentesis incisions were performed using a microvitreoretinal knife (20 gauge) followed by injection of a cohesive ophthalmic viscoelastic device (OVD); injection of sodium hyaluronate ophthalmic solution 1.4% into the anterior chamber; and a clear typical triplanar corneal incision using a 2.2 mm keratome. After forceps-aided capsulorrhexis, hydrodissection was performed and a dispersive viscoelastic fluid, 2% hydroxypropylmethylcellulose, was injected into the anterior chamber for endothelial protection.

**Figure 1:**
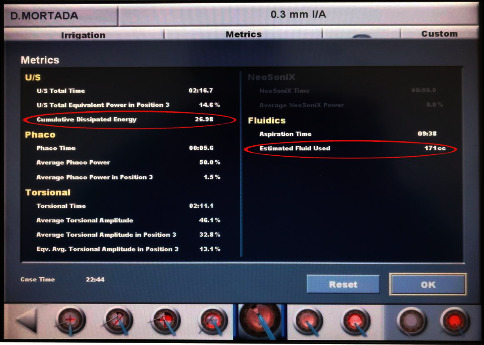
Summary of phacoemulsification parameters at the end of surgery, with the CDE and estimated fluid used highlighted.

Nucleotomy was performed in all cases by using the stop-and-chop technique followed by irrigation-aspiration of the cortical matter and implantation of a foldable intraocular lens. After aspiration of the residual viscoelastic material, the incisions were sealed by hydration without using sutures. Finally, we administered topical moxifloxacin hydrochloride 0.5% eye drops and prednisolone acetate 1% eye drops followed by eye patching. At the end of the surgery, the cumulative dissipated energy (CDE) and estimated fluid used were recorded, as shown in Figure 1.

### 2.3. Postoperative Care and Follow-Up

Patients were instructed to instil moxifloxacin eye drops five times daily for one week and prednisolone eye drops five times daily with gradual withdrawal within four weeks. Slit-lamp examinations were routinely performed during the entire follow-up period. All patients underwent postoperative UCVA, BCVA, subjective refraction, and specular microscopy assessments at 1, 3, and 6 months postoperatively.

### 2.4. Statistical Analysis

Data were analysed using IBM SPSS Statistics for Windows version 20.0. Quantitative data were expressed as mean ± standard deviation, median, and range, while qualitative data were expressed as numbers and percentages. The data were tested for normality using the Shapiro–Wilk test, in which significant results indicated that the findings of nonparametric tests were not normally distributed. Independent-sample *t*-tests and repeated-measures ANOVA with pairwise comparisons were applied for normally distributed data. The nonparametric Mann–Whitney test, Friedman test with pairwise comparisons, and Spearman correlation were used for data that were not normally distributed. The chi-square (*χ*^2^) test and Fisher's exact test were used for the comparison of qualitative variables as appropriate. A 5% level was chosen to indicate significance in all the statistical tests used in the study.

## 3. Results

This retrospective comparative study included 88 eyes from 88 patients (38 men (43.2%) and 50 women (56.8%)) who underwent uneventful phacoemulsification cataract surgery for age-related cataracts (grade N2 or N3). The right eye was operated on in 46 patients (52.3%), while the left eye was operated on in 42 patients (47.7%). The patients were divided into two groups: group A included 31 patients with gout and group B included 57 patients who had no gout or any other systemic disease. Preoperative parameters were matched between the two groups. The mean patient age in group A was 58.23 ± 3.91 years, while that in group B was 59.61 ± 5.99 years. The two groups showed no statistically significant differences in the nuclear grade of cataracts, average keratometry findings, ACD, AL, and IOL power (biometry), as given in Table 1. The specular microscopy values in group A were as follows: mean ECD, 2761 ± 327.17; mean CV, 35.45 ± 4.61; mean HEX, 51.58 ± 8.21; and mean CCT, 512.13 ± 29.22. The corresponding values in group B were as follows: mean ECD, 2844.46 ± 335.1; mean CV, 36.39 ± 4.32; mean HEX, 47.86 ± 7.26; and mean CCT, 515.44 ± 22.9, respectively. In the assessment of intraoperative parameters, the CDE and the estimated fluid used did not differ between the two groups, as given in Table 1.

**Figure 2:**
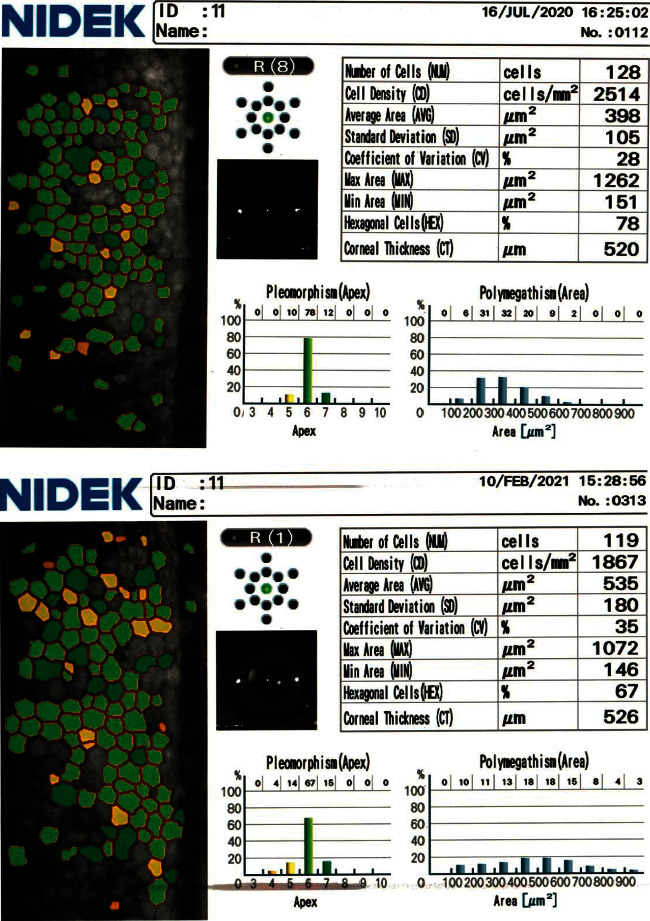
Preoperative and 6-month postoperative specular microscopy findings in a female patient in group A (gout group). The reduction is in ECD and HEX and the increase is in CV and CCT values.

**Figure 3:**
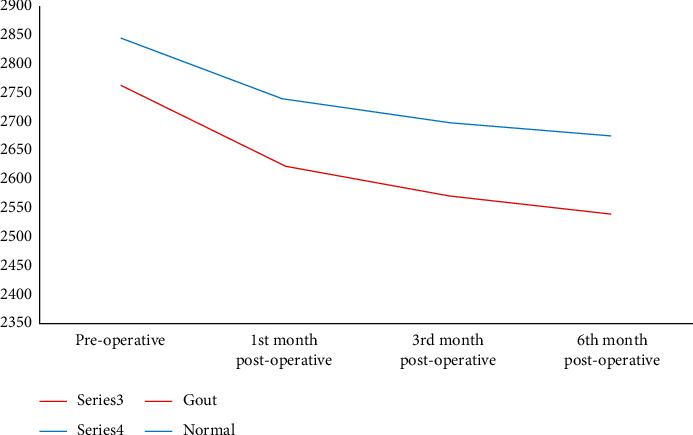
A chart comparing repeated cell density measures in the two groups.

Comparisons of visual acuity before and after surgery in the two groups showed significant improvement in both UCVA and BCVA after the surgery throughout the follow-up period, with no significant differences between the two groups. After surgery, the ECD showed a significant progressive decline in both groups, with mean values of 2622.52 ± 329.08, 2571.06 ± 326.09, and 2539.65 ± 324.7 after 1, 3, and 6 months, respectively, in group A and mean values of 2738.32 ± 342.28, 2697.91 ± 344.41, and 2674.58 ± 342.77, respectively, in group B. This decline was more significant in group A (gout group) than in group B (normal group), with a mean difference of 138.48 ± 41.2, 189.94 ± 50.18, and 221.35 ± 43.87 at 1, 3, and 6 months, respectively, in group A and a mean difference of 106.14 ± 32.62, 146.54 ± 43.3, and 169.88 ± 52.67, respectively, in group B, as shown in Figures 2 and 3 and Table 2. Spearman correlation analysis of the ACD, CDE, and estimated fluid used with the ECD change after the 6^th^ month postoperatively revealed a significant correlation only with ACD (*r* = **−**0.318 and *p* = 0.003).

The other specular microscopic values also showed significant changes after surgery in the two groups, with CV values showing an initial rise followed by a decline, HEX values showing an initial decline followed by an increase, and CCT values showing an initial rise followed by a plateau and a final return to preoperative values. However, the mean differences did not reach statistical significance when the two groups were compared.

With regard to postoperative complications, no differences were noted between the two groups, with posterior capsule opacification (PCO) developing in three cases in each group and macular oedema developing in one case in group A and two cases in group B.

## 4. Discussion

The increasing prevalence of gout may signal the onset of a worldwide epidemic. This condition affects 8.3 million adults in the United States, and its prevalence is expected to increase with the spread of obesity. Considering the increasing use of phacoemulsification surgeries together with the rise in life expectancy and the global increase in gout prevalence, eye surgeons should be aware of the importance of corneal endothelial care in such patients.

In this retrospective comparative study, we evaluated the stress exerted on the corneal endothelium in patients with gout who underwent uneventful phacoemulsification surgery for age-related cataract (group A) and compared them to a control group (group B) with no gout or any other systemic disease.

The two groups showed no differences in their preoperative or intraoperative characteristics. Conversely, the postoperative corneal endothelial characteristics showed significant differences, with more damage to the endothelium observed in the gout group than in the normal group. These differences in the ECD values were significant over the entire follow-up period (6 months) but did not reach statistical significance with regard to CCT, CV, and HEX values.

Sahu et al. [16] studied the effect of uneventful phacoemulsification on the corneal endothelium of 60 patients with controlled diabetes mellitus (type 2 with glycosylated haemoglobin< 7.0) and compared them to the findings in 60 nondiabetic patients. Both groups showed a decline in ECD and HEX and a rise in CV and CCT at the end of the follow-up period of 3 months; however, the changes were more significant in the diabetic group, indicating more endothelial dysfunction. However, this study did not exclude other systemic diseases such as gout from the control group, which may have biased the results.

Kosekahya et al. [17] investigated corneal endothelial changes in 50 patients with gout compared to an age and sex-matched group of healthy individuals. They found that the gout group had a lower mean ECD and HEX and higher CV and CCT than the control group. In addition, they reported a negative correlation between gout duration and both ECD and HEX (*r* = −0.400, *p* = 0.019 and *r* = −0.348, *p* = 0.043, respectively), while the uric acid level was positively correlated with CCT (*r* = 0.355, *p* = 0.003) and negatively correlated with HEX (*r* = −0.245, *p* = 0.044). They concluded that patients with gout might have corneal endothelial dysfunction which is proportional to disease duration and uric acid levels. They urged eye surgeons to consider such endothelial dysfunction when treating patients with cataracts or glaucoma and having gout.

The same group [18] evaluated the characteristics of the cornea and tear film in 41 patients with gout in comparison with healthy individuals. They studied corneal densitometry findings in both groups by using Pentacam corneal tomography and reported significantly higher values in the 0–2 mm and 2–6 mm zones of both anterior and central layers in the gout group compared to the control group, while the densitometric values of the 0–2 mm and 2–6 mm zones of the posterior layer were similar in both groups. The 6–10 mm and 10–12 mm zones of all layers showed similar values in two groups. In addition, they noted a strong positive correlation between the anterior and central 0–2 mm and 2–6 mm zones and both uric acid levels and duration of gout.

To the best of our knowledge, this is the first study to investigate the effects of phacoemulsification cataract surgery on the corneal endothelium in patients with gout. Endothelial cell loss was more severe in patients with gout than in patients without gout or any other systemic disease, indicating the need for greater attention during surgery in such patients.

Hyperuricemia induced in rats was linked to damage of the renal tubular endothelium due to decrease in Na, K-ATPase activity. Higher levels of serum uric acid were associated with a higher decline in enzyme activity [19]. Such mechanisms relating to endothelial dysfunction may also explain the excessive loss of corneal endothelial cells in patients with gout after phacoemulsification cataract surgery.

The main limitations of this study are its retrospective design, small sample size, and relatively short follow-up duration. Although the study included a small number of cases, it can be used as a pilot study to calculate the sample size of more powerful studies. Future randomised controlled clinical trials with larger samples and longer durations of follow-up are needed to confirm the results of this study.

## 5. Conclusions

In conclusion, patients with gout, such as those with diabetes, are probably more prone to corneal endothelial dysfunction postcataract surgery. Thus, surgeries in these patients should be performed using more protective viscoelastic devices or less damaging nuclear-dividing techniques, with more attention to intraoperative phaco parameters.

## Figures and Tables

**Table 1:** Comparison of preoperative and intraoperative characteristics between study groups.

Characteristics	Group		*P* value
	Gout (*N* = 31)	Normal (*N* = 57)	
Gender			<0.001^*∗*^
Female	**27 (87.1%)**	**23 (40.4%)**	
Male	**4 (12.9%)**	**34 (59.6%)**	
Age (year)			0.165
Mean ± S.D.	**58.23** **±** **3.91**	**59.61** **±** **5.99**	
Median (IQ range)	**57 (55–62)**	**60 (55–65)**	
Laterality			0.590^*∗*^
Left	**16 (51.6%)**	**26 (45.6%)**	
Right	**15 (48.4%)**	**31 (54.4%)**	
Grade			0.314^*∗*^
N2	**13 (43.4%)**	**23 (40.4%)**	
N3	**17 (56.6%)**	**34 (59.6%)**	
Preaverage K			0.544
Mean ± S.D.	**45.37** **±** **1.87**	**44.73** **±** **1.42**	
CI of mean	**44.69–46.06**	**44.35–45.11**	
Median (IQ range)	**44.36 (43.75–47.25)**	**45.08 (44.06–45.99)**	
Preaxial L			0.047
Mean ± S.D.	**22.96** **±** **1.07**	**23.77** **±** **1.68**	
CI of mean	**22.56–23.35**	**23.32–24.21**	
Median (IQ range)	**23.11 (22.21–23.6)**	**23.53 (22.37–24.19)**	
Pre-AC depth			0.763
Mean ± S.D.	**3.23** **±** **0.37**	**3.23** **±** **0.32**	
CI of mean	**3.09–3.36**	**3.14–3.31**	
Median (IQ range)	**3.25 (3.06–3.37)**	**3.26 (2.95–3.45)**	
Biometry			0.046
Mean ± S.D.	**20.48** **±** **3.91**	**18.44** **±** **4.45**	
CI of mean	**19.05–21.92**	**17.26–19.62**	
Median (IQ range)	**21 (19–22)**	**20 (18–21)**	
Cumulative dissipated energy CDE			0.389
Mean ± S.D.	**4.62** **±** **3.02**	**6.23** **±** **5.33**	
CI of mean	**3.51–5.73**	**4.82–7.64**	
Median (IQ range)	**4.61 (1.47–6.07)**	**4.19 (3.09–8.53)**	
Estimated fluid used			0.313
Mean ± S.D.	**110.06** **±** **34.19**	**100.04** **±** **28.95**	
CI of mean	**97.52–122.61**	**92.35–107.72**	
Median (IQ range)	**103 (80–141)**	**95 (83–114.5)**	

*P* values were calculated using the Mann–Whitney *U* test. ^*∗*^*P* values were calculated using the Chi-square test. *P* values of <0.05 indicated statistical significance.

**Table 2:** Comparison of changes in study measures between the study groups six months postoperatively.

Characteristics	Group		*P* value
	Gout (*N* = 31)	Normal (*N* = 57)	
UCVA			0.608
Mean ± S.D.	**−0.48** **±** **0.2**	**−0.5** **±** **0.22**	
CI of mean	**−0.56–0.41**	**−0.56–0.44**	
Median (IQ range)	**−0.45 (−0.57–0.4)**	**−0.45 (−0.62–0.4)**	
BCVA			0.492
Mean ± S.D.	**−0.62** **±** **0.24**	**−0.64** **±** **0.23**	
CI of mean	**−0.71–0.53**	**−0.70–0.58**	
Median (IQ range)	**−0.58 (−0.9–0.4)**	**−0.58 (−0.9–0.47)**	
Cell density			<0.001
Mean ± S.D.	**221.35** **±** **43.87**	**169.88** **±** **52.67**	
CI of mean	**205.26–237.45**	**155.90–183.85**	
Median (IQ range)	**210 (198–255)**	**172 (148–191)**	
CV			0.03
Mean ± S.D.	**−1.77** **±** **2.92**	**−3.46** **±** **4.34**	
CI of mean	**−2.84–0.70**	**−4.61–2.30**	
Median (IQ range)	**−1 (−3–0)**	**−3 (−6–1)**	
Hex			0.82
Mean ± S.D.	**5.45** **±** **4.21**	**5.72** **±** **5.62**	
CI of mean	**3.91–7**	**4.23–7.21**	
Median (IQ range)	**5 (2–8)**	**5 (2–9)**	
CCT			0.234
Mean ± S.D.	**−2.65** **±** **6.75**	**−1.39** **±** **14.17**	
CI of mean	**−5.12–0.17**	**−5.15–2.37**	
Median (IQ range)	**−1 (−7–2)**	**−1 (−7–5.5)**	

*P* values were calculated by the Mann–Whitney *U* test. *P* values of <0.05 indicated statistical significance.

## Data Availability

The datasets used and/or analysed during the current study are available from the corresponding author upon request.
